# Mixed Reality-Based Interaction between Human and Virtual Cat for Mental Stress Management

**DOI:** 10.3390/s22031159

**Published:** 2022-02-03

**Authors:** Heewon Na, Soyeon Park, Suh-Yeon Dong

**Affiliations:** Department of Information Technology Engineering, Sookmyung Women’s University, Seoul 04310, Korea; anny505@sookmyung.ac.kr (H.N.); skygreen97@gmail.com (S.P.)

**Keywords:** human–animal interaction, mixed reality, mental stress relief, virtual animal

## Abstract

Human–animal interaction (HAI) has been observed to effectively reduce stress and induce positive emotions owing to the process of directly petting and interacting with animals. Interaction with virtual animals has recently emerged as an alternative due to the limitations in general physical interactions, both due to the COVID-19 pandemic and, more generally, due to the difficulties involved in providing adequate care for animals. This study proposes mixed reality (MR)-based human–animal interaction content along with presenting the experimental verification of its effect on the reduction of mental stress. A mental arithmetic task was employed to induce acute mental stress, which was followed by either MR content, in which a participant interacted with virtual animals via gestures and voice commands, or a slide show of animal images. During the experiment, an electrocardiogram (ECG) was continuously recorded with a patch-type, wireless ECG sensor on the chest of the subject, and their psychological state was evaluated with the help of questionnaires after each task. The findings of the study demonstrate that the MR-based interaction with virtual animals significantly reduces mental stress and induces positive emotions. We expect that this study could provide a basis for the widespread use of MR-based content in the field of mental health.

## 1. Introduction

University students are generally faced with various stressful situations and concerns. Some of the common sources of stress for university students include new environments, academic overload, pressures for employment and success, competition with peers, and worries about the future. Such extreme stress lowers the students’ quality of life and deteriorates their health, which leads to maladjustment to school life, adversely affects the academic performance, and results in various negative psychological and physical experiences [1]. However, most university students undergo treatment only after their symptoms worsen, and are generally unaware of stress or depression. Therefore, coping with the stress is crucial in improving the mental health of university students.

Over the past 20 years, several studies have been conducted to measure the therapeutic value aspects of being with animals and human–animal interactions (HAIs). In a previous study, it was found that, compared with those without companion animals, the heart rate and blood pressure levels of people with companion animals were significantly lower, they showed less stress response even in stressful situations, and a faster recovery was observed [2]. This revealed that people perceive companion animals as an important part of their lives, and significant cardiovascular and behavioral benefits are associated with this perception. Another study found that being with a new dog reduced cortisol and heart rate during stress tests compared with being with human companions, suggesting that short-term exposure to a new dog, even in an unfamiliar environment, may be beneficial [3]. Furthermore, interactions with animals have been shown to reduce anxiety, loneliness, depression, and stress-related parameters, such as cardiovascular disease, in humans [4,5]. Therefore, HAIs are expected to play a key role in the areas of psychological and psychophysiological well-being. Currently, nearly 1000 university campuses in the USA have established the Animal Visitation Program (AVP), which aims to reduce student stress through human–animal interactions, giving the students an opportunity to pet and interact with animals [6]. In previous studies, the AVP has enabled students to pet cats and dogs, providing momentary stress relief [7], reducing perceived stress [8], increasing positive emotion levels, and decreasing negative emotion levels [9].

However, one study has demonstrated that HAI increases stress behaviors in animals, such as dogs licking their lips or yawning [10]. Thus, while HAI can reduce stress in humans, it can also cause increased stress in animals. Additionally, several limitations exist in supporting HAI in universities, such as animal allergies, safety issues, and difficulty in providing the necessary space. Moreover, the recent COVID-19 pandemic has increased the difficulties in interactions with animals, as well as between humans. Therefore, interactions with virtual animals instead of real ones can be considered as an alternative to overcome these limitations. Norouzi’s recent paper [11] states that virtual animals have already been introduced through pet games, such as Tamagochi (https://tamagotchi.com/ accessed on 3 August 2020)and Nintendo games, such as “Nintendogs + cats: Golden Retriever & New Friends” (https://www.nintendo.com/games/detail/nintendogs-cats-golden-retriever-and-new-friends-3ds/ accessed on 3 August 2020). Lin et al. also reported that pet games provide emotional support through pet interaction, without involving animal-related issues, such as allergies [12]. Furthermore, research indicates that interactions with virtual animals improve the academic performance of students and have a positive effect on their physical activities [13,14,15].

With the development of virtual reality, frameworks are being developed that can simulate situations that cannot be easily engaged with in reality, providing users with a high sense of reality and smooth interactions [16]. Furthermore, in contrast to existing entertainment platforms, realization of augmented reality (AR) and mixed reality (MR) techniques can enable the virtual animals to be engaged with in the real world, sharing the same physical space as that of humans. The users can see and interact with virtual animals in the real world with the help of MR glasses. Additionally, the graphical representation of the MR animals provides a more realistic experience to the users when compared with robots and desktops used thus far [17]. However, limited research has been conducted to verify whether MR-based virtual animals have a positive effect on the mental health of human users.

Various parts of the body contribute to the response to stress, among these, heart rate variability (HRV) is widely used for the objective evaluation of psychological health and stress [18,19]. Mental work (stress) and the subsequent recovery and rest affect the HRV, which corresponds to the autonomic nervous system (ANS). Therefore, the HRV acts as a powerful tool for observing the interactions between the sympathetic and the parasympathetic nervous systems, and is widely accepted to reflect ANS activity [20,21]. Therefore, this study aims to implement the MR-based HAI and to experimentally verify, through HRV analysis, whether it is effective in relieving stress in university students.

## 2. Program Design and Implementation

### 2.1. Platform Overview

Microsoft HoloLens (Microsoft, Redmond, WA, USA) ( https://www.microsoft.com/en-us/hololens accessed on 1 September 2020) is widely used to implement MR content. HoloLens (1st Gen) is a head-mounted device that can recognize and utilize the surrounding environment through a spatial feature point extraction method [22]. It also supports gesture input using fingers, as well as voice input. Developing content for HoloLens requires the use of the Universal Windows Platform (UWP). Unity supports the UWP and is widely used by developers and researchers because of its fidelity, physics simulation capabilities, accessibility, and community support [23,24]. Furthermore, Microsoft recently restructured the Mixed Reality Tool Kit (MRTK), which is useful in developing applications for the HoloLens [25]. MRTK v2.4.0 is used in this study, and the animation control and the interaction configuration of virtual animals within Unity are implemented in C# using Visual Studio 2019.

### 2.2. Animations

**Figure 1:**
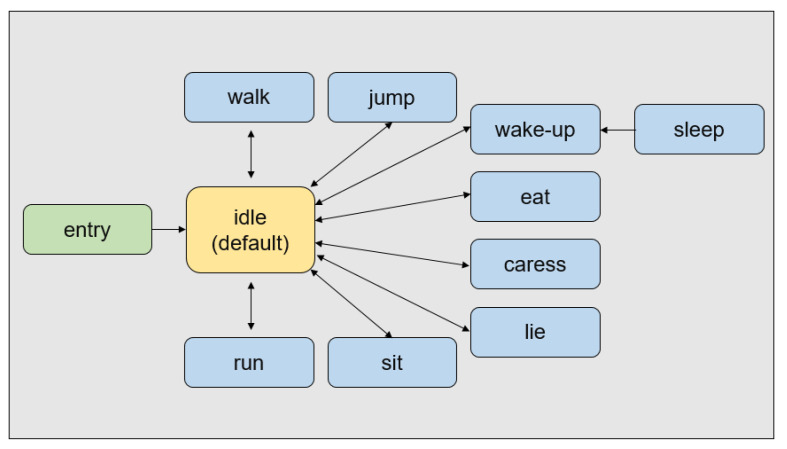
Animator configuration.

**Figure 2:**
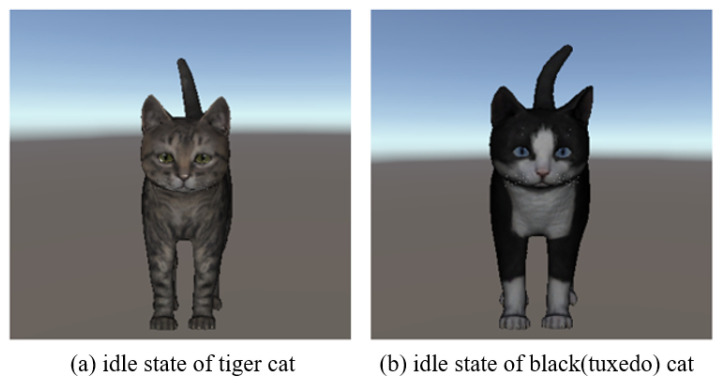
Idle state of virtual cats.

Schwind et al. stated that people feel more comfortable and interact better with the virtual cat model if the quality of the model is higher and if it looks more natural and less intimidating [26]. Therefore, a rigged kitten (short) (https://assetstore.unity.com/packages/3d/characters/animals/mammals/kitten-short-132026 accessed on 10 September 2020) model that was animated and rendered via the Unity graphics engine was used in this study. The cat model includes three different textures (black(tuxedo), gray, and tiger) and various animated behaviors, such as sitting, running, and crouching, allowing for the developers to change the appearance of the cat and control its behavior. In this study, eight different behaviors are configured: idle, eating, walking, running, sitting, sleeping, jumping, lying down, and caress (see Figure 1). The idle state is maintained as the default state, as shown in Figure 2, and a bool-type parameter was created for each animation to connect the transition with the idle state to be controlled. The parameter value was initialized as false to ensure that the idle state is maintained initially. The animation is activated if the animation’s parameter value becomes true under the user’s control. After the animation, the parameter value of the specific animation returns to false, and the animal’s state returns to idle. The transition, connected in the direction of entering the idle state, has a fixed exit time to ensure that the motion of the virtual cat is not interrupted. The transition to the idle state occurs when the user wakes up the virtual cat only in the sleeping state.

### 2.3. Implementation of Human—Animal Interactions

#### 2.3.1. Gestures

**Figure 3:**
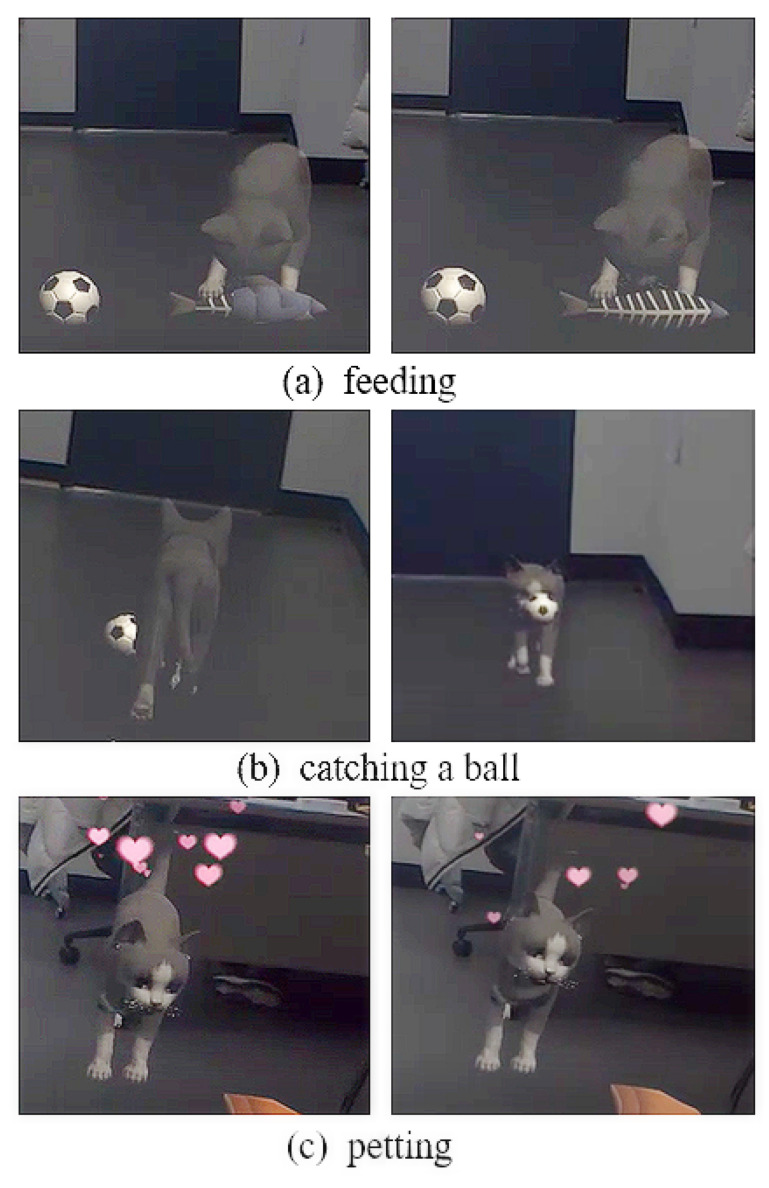
Examples of interaction with gestures.

A profile of the HoloLens 1 version of MRTK was created and an input action handler is used to recognize the user’s gesture. The interaction through gestures consists of catching a ball, feeding, and petting, as shown in Figure 3. Initially, when the user air-taps the ball, the ball rolls, the cat chases the ball and bites it, and then brings it to the user, as shown in Figure 3a. The air-tap gesture is achieved by holding a fist, such that the back of the user’s right hand faces toward the body and the second finger is gently bent, as if tapping the air. This gesture is recognized as “Select” in the MRTK Input Action Handler. Consequently, the ball is made to roll by activating the code, which controls the rotation, the position, and the movement speed of the ball. The virtual cat is rotated in the direction of the ball and the position vector is moved toward the position vector of the ball, corresponding to the change in the position of the ball. The cat is brought back to the user while holding the ball as soon as the position vector of the cat matches with that of the ball, and the ball is deactivated when the cat returns to its original position. While the virtual cat is moving, the running state of the animation is activated to produce a running motion.

Subsequently, when the user taps on the fish or the ham, the virtual cat is configured to eat. The fish and the ham are composed of four parts, and the IEnumerator is used to assign a time term and deactivate each part sequentially, expressing the cat gradually eating the ham and the fish (Figure 3b). The virtual cat bows its head to produce an eating animation and returns to the idle state after only the bones of the ham or the fish remain. Additionally, after the cat is finished eating, it produces a star-shaped particle effect to indicate that the cat likes the food.

Lastly, the user can pet the cat. When the cat is idle, air-tapping the cat instantly activates the caress animation to show its charm. At this time, a heart-shaped particle effect was added to express the cat’s happiness (Figure 3c).

#### 2.3.2. Voice Commands

**Figure 4:**
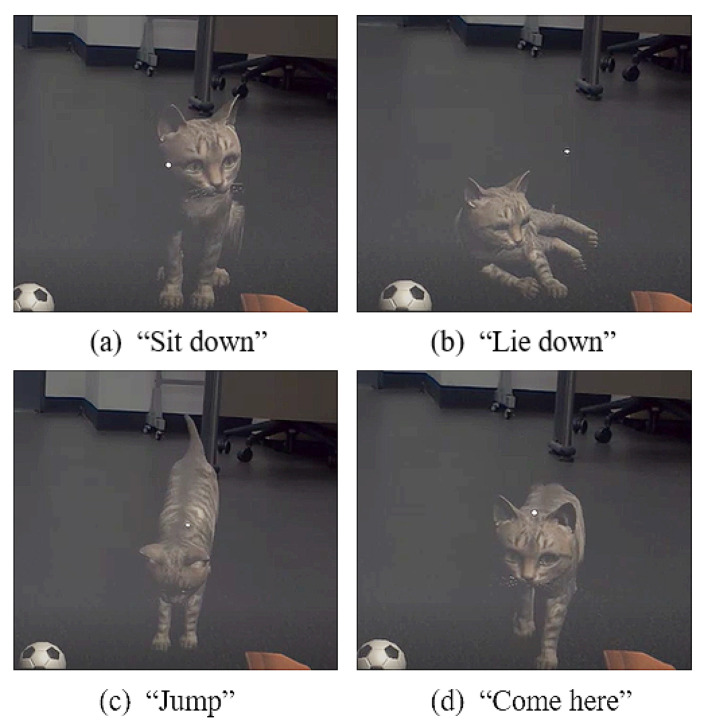
Examples of interaction with voice commands.

The GameVoiceControl plugin (https://assetstore.unity.com/packages/tools/audio/game-voice-control-offline-speech-recognition-178047 accessed on 10 September 2020) is used to recognize the voice of the user. Four types of voice commands and interactions were organized in this study, as shown in Figure 4. The GameVoiceControl prefab is loaded into the hierarchy, the language is set to English, and Textlog is connected and registered in Commands to recognize four words: “Jump”, “Sit down”, “Lie down”, and “Come here”. The words spoken by the user are then displayed and recognized in the text object, and when the voice command matches one of the four commands, the animation corresponding to that command is activated. When the motion is completed, the animation is deactivated again, and it returns to the idle state.

## 3. Experiment Methods and Results

### 3.1. Methods

The experiment involved 30 healthy university students (mean (M) ± standard deviation (SD) age 21.7 ± 1.44). Students with a history of heart disease or neuropsychiatric disorders, those with current medications, and pregnant or prospective women, were excluded from the study. Additionally, the subjects who generally wear glasses were asked to wear contact lenses while participating in the experiment, in order to wear the Hololens. All the experimental procedures involving human subjects were approved by the institutional review board at the affiliated institution. The entire experimental procedure was verbally introduced to the participants, and their written informed consent was obtained.

#### 3.1.1. Experimental Design

**Figure 5:**
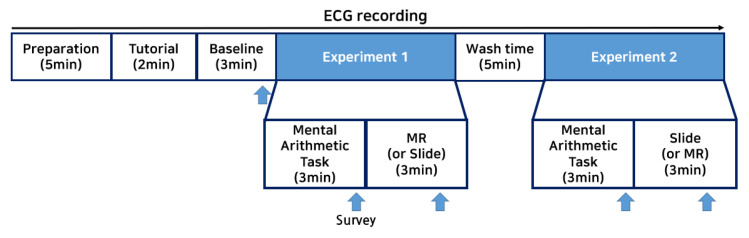
Experimental procedure.

**Figure 6:**
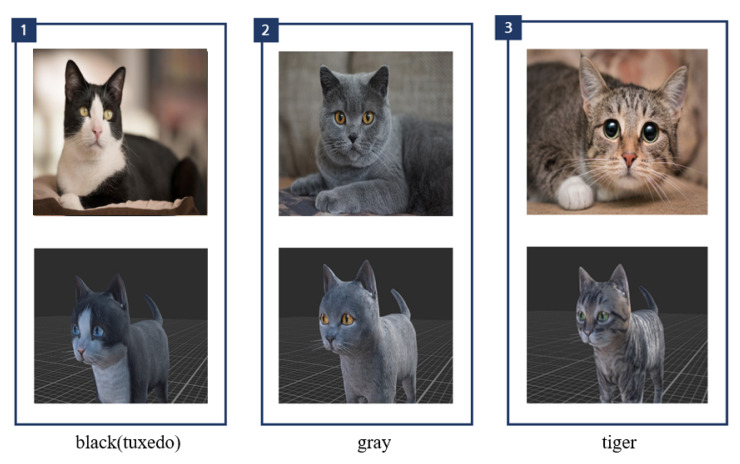
Animal image examples.

This study was conducted with a series of steps, in the following order: “Preparation”–“Tutorial”–“Baseline”–“Experiment 1”–“Wash time”–“Experiment 2” (see Figure 5). The total time required was approximately 35 min. Initially, the subject was shown a few pictures of cats (as shown in Figure 6) and was instructed to choose the most preferred in terms of the appearance. The cat selected by the subject was used during the subsequent experiment.

An electrocardiogram (ECG) sensor was attached to each subject’s chest to continuously measure their ECG from the stage of wearing the equipment to the end of the experiment. In this study, every subject was asked to wear a patch-type ECG sensor (T-REX, Taewoong Medical Inc., Gimpo-si, Gyeonggi-do, Korea) on the chest. This sensor is a square-shaped ECG sensor in the form of a detachable patch, used to measure the electrical activity of the heart in a non-invasive way. In the Preparation step, the subjects were provided with the details of attaching the sensor to their chests by themselves. The subjects were asked to wear it during the entire course of the experiment to continuously measure the changes registered by the ECG during the experiment. Questionnaire surveys were conducted five times, in the baseline measurement, to measure the subject’s psychological changes after the tasks of Experiment 1 and Experiment 2.

**Figure 7:**
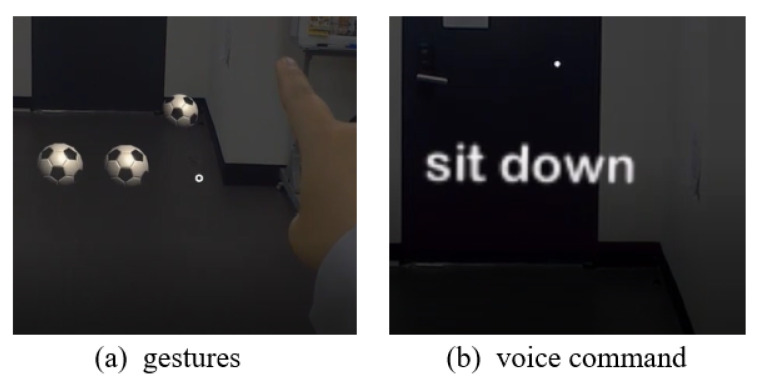
Examples of tutorial.

A tutorial was conducted after wearing the HoloLens to familiarize the subject with the MR environment, the gestures, and the voice commands. As shown in Figure 7, the subjects went through the acclimatization process by rolling the ball using hand gestures and speaking words into the microphone to verify the functionality of the voice recognition system. The tutorial was repeated until the experimenter determined that the subject was sufficiently familiarized with it. The subject was then asked to sit still for 3 min to measure their ECG baseline. The survey was conducted after the baseline measurement was completed.

**Figure 8:**
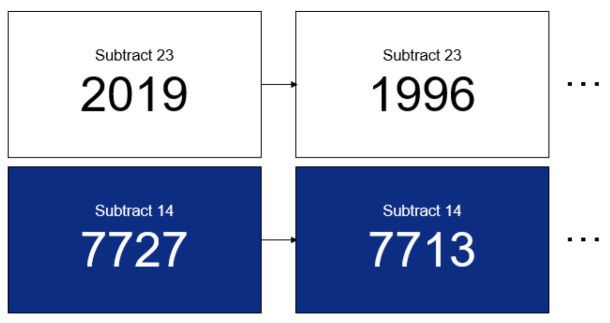
Examples of Mental Arithmetic Task.

In both Experiment 1 and 2, there were sub-steps: a “Mental Arithmetic (MA) task”, a “Questionnaire”, the “MR content experience or watching a slide show”, and a “Questionnaire”. The MA task was conducted to induce mental stress in the subjects, since it is a well-known and established stressor, described as a psychosocial stress factor because it includes both cognitive (i.e., subtraction tasks) and social (i.e., performing a task in front of an experimenter) factors, which generate stress responses [27]. Since there were two MA tasks for each subject, the MA task performed before the MR content was termed MA1, and the MA task performed before the slide show was termed MA2. The subjects were instructed to answer correctly after seeing a four-digit number in the center of a flat screen and subtracting a constant two-digit number that appeared above the four-digit number. The MA task was carried out for three minutes, and the subtraction continued until the four-digit number changed on the screen; the four-digit number changed every minute. For example, as shown in Figure 8, a 4-digit number, 2019, is displayed on the screen, and the user is required to answer aloud by subtracting 23 consecutively. When subjects say the result of the subtraction, the number with the subtraction applied appeared on the screen—1996 (=2019 − 23) in this example. Subjects naturally knew whether the number they answered was correct or not by looking at the numbers on the next screen. After a minute, a new 4-digit number, 7727, for example, appeared on the screen, and the users were instructed to answer by subtracting 14 consecutively. Since the subtraction speed was different for each subject after every minute, the background color was different for classification when changing to a new four-digit number. The four-digit numbers used in this study were randomly generated to ensure fair difficulty among the subjects.

After completing the MA task, the subjects responded to the questionnaire and then either performed the HAI task with virtual cats or watched a slide show consisting of still images of the cats for three minutes. The length of the experimental session was set close to the average time when all the functions of the MR content were repeated 1 or 2 times—evaluated through a pilot test. These two different tasks were conducted in a random order for Experiment 1 and 2, respectively. Some of the subjects performed the HAI in Experiment 1 and watched a slide show in Experiment 2, while other subjects watched a slide show in Experiment1 and performed the HAI in Experiment 2. The slide show displayed each image of the animal for five seconds and ran for a total of three minutes. In a previous study, looking at photos of personal pets or unfamiliar animals while performing a mental arithmetic task made people feel more comfortable; although, these results indicated no less stress than looking at photos of a supportive important person or an unfamiliar person; they also observed that active interaction with pets is necessary to reduce stress [28]. Therefore, in the present experiment, a slide show of animal images was chosen as a control for the MR content interacting with virtual animals. The “Wash time” step was carried out to eliminate the effect of the previous stimulation and the subject was asked to sit in a chair for five minutes and rest comfortably.

#### 3.1.2. Electrocardiogram (ECG) Recording

In this paper, the mean heart rate (HR—average heart rate) in the frequency domain, the HFnu (high-frequency normalized units; the intensity of the frequency band corresponding to 0.15–0.4 Hz), and the LF/HF ratio (ratio between LF and HF) parameters were selected in the time domain.

The state in which stress is not recognized is called the recovery state, which is defined as the parasympathetic dominance state of the ANS. Therefore, the variables related to the parasympathetic dominance of the ANS are used to detect recovery. These include the HF and the HR. Recovery generally takes place during sleep, relaxation, rest, or other stress-free tasks, where the overall physical activity is low. In the recovery state, the HR is individually low and the HRV is large and uniform. The higher the HF, the more active the parasympathetic nervous system is, thus indicating a recovery state [29]. Conversely, under stress conditions, the sympathetic nervous system activity is dominant, and when the parasympathetic activation is low, it is defined as an increase in the body activation. The variables related to the sympathetic dominance of the ANS are used for stress detection. In a state of stress, an individual’s HR rises and implies a state of tension. Additionally, the LF/HF reflects the overall balance of the sympathetic and the parasympathetic nerves, or the autonomic nerves. In certain cases, it is also used as an indicator of the sympathetic nerve activity. The LF/HF is proportional to the sympathetic activity and is inversely proportional to the parasympathetic activity. Furthermore, a decrease in the high-frequency component and an increase in the low-frequency component of the HRV are observed during psychological stress, resulting in a large increase in the LF/HF ratio [30].

#### 3.1.3. Survey

A total of five surveys were conducted to monitor the psychological state changes of the subjects. The POMS (Profile of Mood States) is a clinically used questionnaire which evaluates temporary emotions and moods, and has been widely used to diagnose various emotions and moods [31]. In this study, a short Korean version of the POMS, known as the K-POMS-B, was used, which consists of a 5-point scale of 30 items [32]. The POMS is composed of six sub-areas—tension and anxiety (T-A), depression (D), anger and hostility (A-H), vigor (V), fatigue (F), and confusion (C)—and the scores can be calculated per sub-area. As observed in Table 1, a total of 29 items—excluding 1 item, “forgetting well” which did not fit the requirements of the experiment—in the existing K-POMS-B were used; additionally, the current stress level was verified on a scale of 1–5. The total mood disturbance (TMD) can be calculated by subtracting the score of the vigor, which is a positive mood measure, from the sum of the scores of the remaining five areas, which are negative mood scales. Higher TMD scores indicate greater negative emotions.

### 3.2. Experiment Results

#### 3.2.1. Electrocardiogram (ECG)

In this study, some HRVs were considered, to measure the physiological responses during the experiment. Each parameter measurement was calculated and compared at 1 min intervals for each stage of the experiment, according to the literature related to ultra-short-term HRV analysis in mental stress detection [33,34]. A total of 30 samples were used in this experiment, and the Friedman Test was performed on 5 task scores, along with a post-test measure, because the normality was not satisfied. Previous studies list several approaches for statistical inference in different contexts, and recommended the Friedman test paired with the post hoc Nemenyi test for reasoning about comparisons between multiple groups on multiple data sets [35]. Therefore, if the Friedman test indicated significance, we performed the Nemenyi post hoc test.

**Figure 9:**
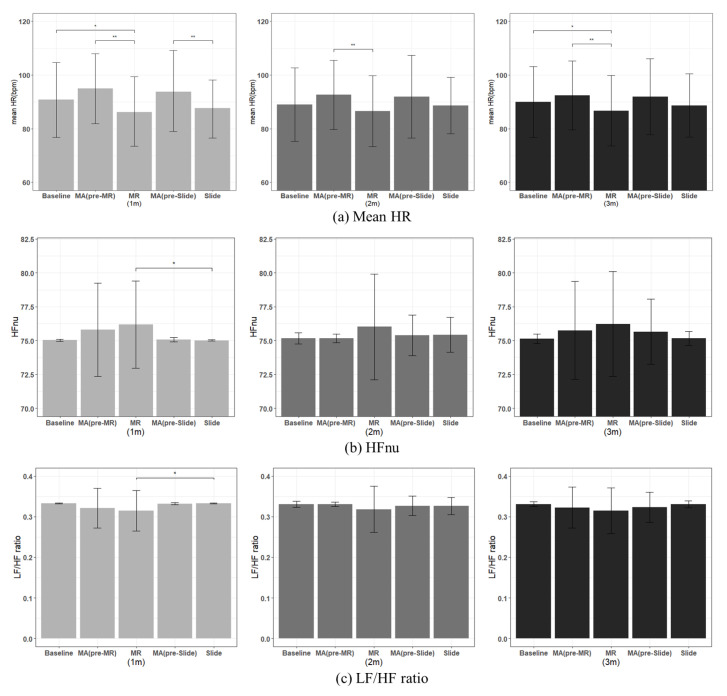
Comparison of mean HR and HRV results between average of baseline, MA (pre-MR), MR, MA (pre-slide), and Slide (the slide show). N = 30, mean ± standard error, *: *p* < 0.05, **: *p* < 0.01; significant differences verified using Friedman test, Nemenyi post hoc test.

Firstly, the mean HR of the MR was significantly lower in all the intervals when compared with the mean HR of the MA (pre-MR), as shown in Figure 9a and Table 2. The slide mean HR was significantly lower than the MA (pre-MR) in the 1-min section. Essentially, when performing the MR contents or while watching a slide show after the mental arithmetic process, which is a stressful situation, the heart rate was significantly decreased. Furthermore, in the case of the MR, the average heart rate was significantly lower in the 1-min and 3-min sections when compared with the baseline, unlike the slide show. When comparing the MR and the slide show, the MR tended to be generally lower than the slide show, but not by a significant amount.

In the case of the HF, which is an activation index of the parasympathetic nervous system, the HF of the MR was significantly higher than that of Slide during the first minute. The LF/HF ratio of the MR, which was used as an activation index of the sympathetic nervous system, was significantly lower during the first minute compared with the slide. Furthermore, although not by significant amount, the HF was higher and the LF/HF ratio was lower, when compared with the MA (pre-MR) in all sections. Conversely, the slide show had lower HF and higher LF/HF ratios when compared with the MA (pre-Slide) in the 1 min and 3 min sections (Table 2).

#### 3.2.2. Survey

**Figure 10:**
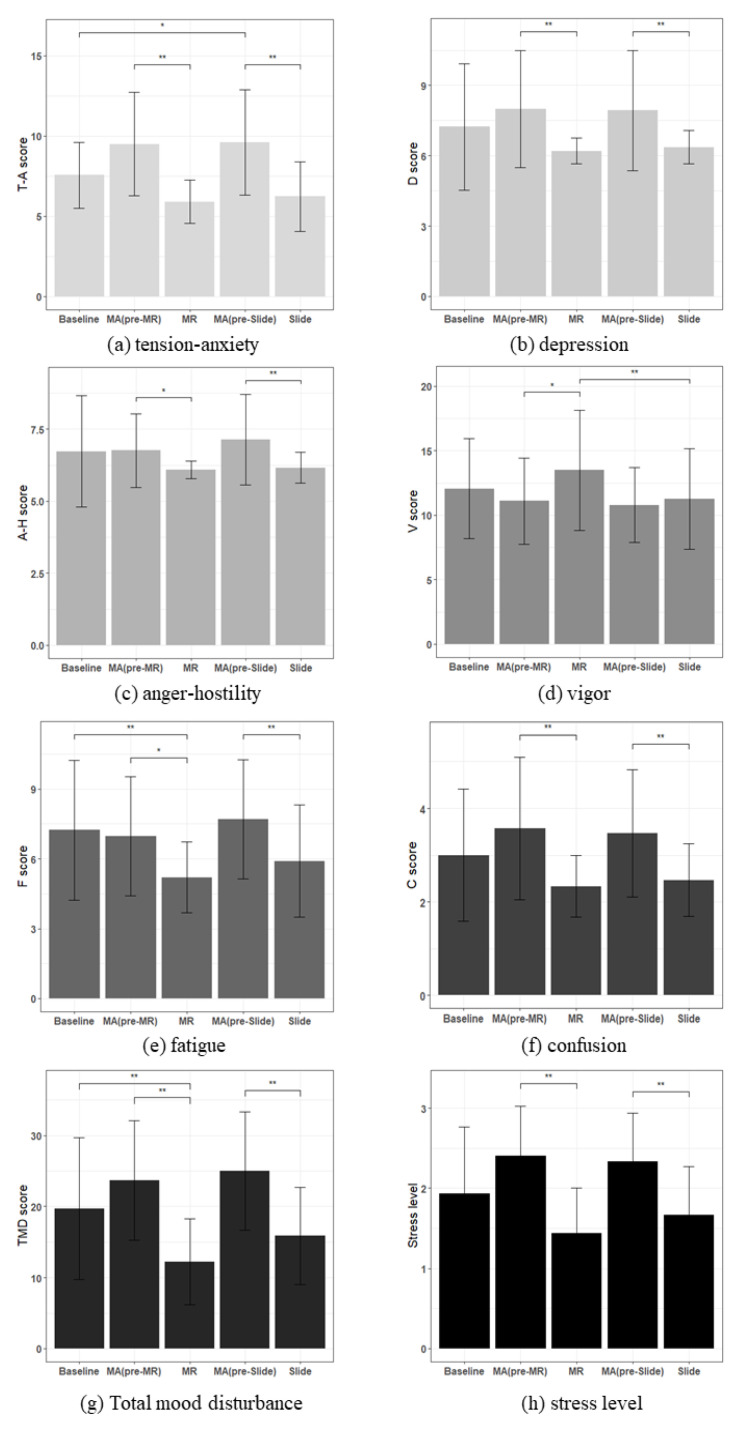
Comparison of survey result between average of baseline, MA (pre-MR), MR, MA (pre-slide), and Slide. N = 30, mean ± standard error; *: *p* < 0.05; **: *p* < 0.01; significant differences verified using Friedman Test, Nemenyi post hoc test.

Through the K-POMS-B questionnaire, mood status was evaluated immediately after each task by scoring approximately six sub-areas and the TMD. The psychological evaluation also included the Friedman test and Nemenyi post-test for five task scores (Table 3). In the case of tension–anxiety, the MR and Slide were significantly lower than the MA (Figure 10a). The depression score was significantly lower in the MR and Slide than in the MA (Figure 10b). Anger–hostility was also significantly lower in the MR and Slide than in the MA (Figure 10c). The vigor score, indicating positive emotion, was significantly higher in MR than MA and Slide (Figure 10d). The fatigue scores of MR and Slide were significantly lower than the MA, and the MR was significantly lower than the baseline as well as the MA (Figure 10e). In the case of confusion, the MR and Slide were significantly lower than that of MA (Figure 10f). The TMD score was significantly lower than the MA and the baseline in the MR, and significantly lower than the MA in Slide (Figure 10g). Lastly, in the case of the stress level, the MR and Slide were significantly lower than that of the MA (Figure 10h). Therefore, the MR and Slide can effectively reduce negative emotions. Additionally, in fatigue and TMD, unlike Slide, the MR was significantly lower than the baseline, and MR scores were the lowest in all the negative items and the highest in vigor. Furthermore, MR was significantly higher in the vigor score compared with the slide show. Therefore, it can be said that MR increased positive emotions more than the slide show.

## 4. Discussion

This study aimed to analyze the effect of stress reduction using an MR-based HAI. The experiment was conducted with a total of 30 healthy university student subjects. Mental arithmetic tasks were provided to experimentally induce mental stress in the subjects. Subsequently, stress reduction associated with either an MR-based interaction with virtual animals or a slide show of still images of animals were compared. A questionnaire survey and the heart rate variability using a single-lead ECG on the chest were used to quantitatively compare the stress reduction effect.

Firstly, some of the HRVs analyzed in this study demonstrated significant changes due to stress and recovery because of the ECG recording. The mean HR decreased significantly in the MR when compared with the MA; this result was consistent with the general findings of the lower HR during recovery in ECG studies [36]. The slide show had a significantly lower mean HR than MA in the first minute section. The MR condition led to significantly reduced HR when compared to the baseline, indicating that it effectively relieved tension. Additionally, the HF in the MR was significantly higher in the first minute section than the slide show and was also higher than the baseline and the MA. This result was consistent with previous studies, which reported a decreasing trend in the HF during stress [37,38]. The HF is an indicator that adequately reflects the activity of the parasympathetic nervous system, and the increase in the HF is due to slow deep breathing, which is closely related to the respiratory cycle [39]. Therefore, the increase in the HF can be interpreted as a steady state, due to the MR experience. Similarly, the LF/HF, which is inversely proportional to the activity of the parasympathetic nervous system, exhibited significantly lower MR in the first minute section when compared with the slide show and was also lower than the baseline and MA. This is identical to the previous studies, which show the increase or the decrease in the LF/HF ratio function as self-reported stress, which changes throughout the day [40]. Consequently, tension can be relieved by reducing the heart rate, which is achieved by removing the subject from the stressful situation, but it is observed that the MR experience further reduced the heart rate and increased the parasympathetic nervous system activity. In addition, the standard error of HRV values differed significantly between MR and the others at the 1- and 2-min intervals. It seems that the degree of activation of the sympathetic/parasympathetic nervous system may be different depending on the subject’s familiarity with MR content, recognition of MR motion, and animal preference. In future experiments, additional information related to personal MR experiences and pet experiences may help to understand these differences.

Secondly, scores were calculated for each sub-area for each test to calculate positive or negative emotions, through the K-POMS-B, and the TMD was also calculated to compare the values. All scores representing negative emotions were significantly reduced in MR and slide show than when doing mental arithmetic tasks—a stressful situation. Additionally, in the scores associated with fatigue and the TMD, MR was significantly higher than the baseline, unlike the slide show. This suggests that fatigue and total mood disturbance may be improved if MR content is engaged with during normal times rather than stressful situations. The scores representing positive emotions (the sub-region V) were significantly higher on MR than MA and slide show, suggesting that interaction experiences with virtual animals may help to induce positive emotions in subjects. Therefore, it is suggested that MR and the slide show are both effective in reducing stress, but unlike the slide show, MR is also effective in increasing positive emotions. Considering this, together with the HRV results, it can be said that the subtle difference between the slide show and MR, which was found at the 1 min interval, while less than the difference between MA and MR (or the slide show), might provide evidence that these differences are in the same vein. As a result, the MR-based HAI can be used to reduce mental stress in university students by easing negative emotions and enhancing vitality when interacting with virtual animals.

Subjective feedback was also received after the experiment, corresponding to the MR contents and the interaction experience. The subjects felt like they were interacting with a real cat in the real world; however, there were also opinions that it would be more realistic if the virtual cat could make a sound. There was also an opinion that the possibility of using this content to relieve the stress of university students was high. Some of the subjects had never experienced VR or MR content before the present study, but said that the content was very novel and highly interesting. Future studies will include physiological assessments by some HRVs and also use a wider variety of physiological assessment indicators to support the results. In this study, experiments were conducted only with virtual cats, not with various virtual animals. Since the results may vary depending on the familiarity or appearance of the virtual animal, we plan to conduct experiments using various virtual animals in the future. As mentioned in the Introduction, MR was suggested as a supplementary measure to overcome human–animal interaction, because animals can be stressed by such interactions, and there are other problems, such as allergies and safety issues. However, it is an important point to note that interactions with real animals and interactions with virtual animals have significant differences in stress levels. Future research also aims to solve the problem of interaction with real animals and compare them with virtual animals. Furthermore, in order to intensively prove the effect of “interaction” with virtual animals, it is necessary to compare them with moving animal videos or MR content without interaction. This is because the difference from the slide show can be affected not only by interaction but also by the movement of animals. It is also necessary to conduct research assessing measures of novelty and inquiry spirit that are expected to be stimulated by the MR technology. Additionally, the MR contents will be enhanced significantly and realistically, by configuring virtual animals with different behavior patterns depending on the user’s stress level, biorhythms, and the surrounding environment, as well as interactions based on the user’s preceding behavior.

## 5. Conclusions

In this study, we examined the contents of interactions between humans and virtual animals using an MR-based experience—one of the key technologies of the 4th industrial revolution—supplementing the limitations encountered when interacting with real animals. With this technology, users can easily interact with virtual animals through gestures or voice commands, without the requirement for a controller. The study is also of great significance because it confirmed that the stress of university students was relieved while experiencing the MR content, when compared with viewing a slide show of actual animal images. This was assessed using physiological indicators and psychological questionnaires. Additionally, only experiencing the MR for a short period of time reduced negative emotions of the subjects and increased their positive emotions; this result demonstrates that this technology can significantly reduce mental stress among university students. One drawback experienced by users was that the experience of interacting with the virtual cat could be made more realistic by including sounds. The results of this study prove the positive effect of interaction with virtual animals on the stress control of university students, and might contribute to a more widespread use of MR-based human–animal interactions in the field of mental health.

## Figures and Tables

**Table 1:** K-POMS-B.

Factors	Item	Factors	Item
T	Nervous	A	Angry
F	Worn out	V	Lively
C	Confused	T	On edge
D	Sad	V	Active
A	Annoyed	V	Energetic
D	Miserable	T	Panicky
F	Tired	A	Bitter
D	Downhearted	T	Worried
D	Worthless	C	Mixed-up
F	Exhausted	T	Anxious
D	Depressed	F	Sleepy
A	Furious	D	Discouraged
A	Resentful	V	Competent
V	Vigorous	A	Bad tempered
V	Full of pep		

**Table 2:** ECG result for each parameter—mean (SD), superscripts indicate significant differences across columns; * *p* < 0.05; ** *p* < 0.01; a—baseline; b—MA (pre-MR); c—MR; d—MA (pre-slide); e—Slide.

		Baseline	MA (Pre-MR)	MR	MA (Pre-Slide)	Slide
		a	b	c	d	e
	1 m	90.77 (14.02) ^c^*	95.03 (13.10) ^c^**	86.01 (12.69) ^a^*^b^**	93.77 (14.80) ^e^**	87.62 (11.04) ^d^**
Mean HR (bpm)	2 m	89.02 (13.74)	92.65 (12.91) ^c^**	86.25 (13.24) ^b^**	91.87 (15.39)	88.67 (10.53)
	3 m	90.0 (13.18) ^c^*	92.38 (12.83) ^c^**	86.74 (13.13) ^a^*^b^**	91.97 (14.15)	88.69 (11.81)
	1 m	75.04 (0.08)	75.80 (3.43)	76.19 (3.23) ^e^*	75.07 (0.15)	75.02 (0.04) ^c^*
HFnu (%)	2 m	75.16 (0.42)	75.16 (0.32)	76.02 (3.91)	75.39 (1.49)	75.43 (1.29)
	3 m	75.14 (0.34)	75.75 (3.60)	76.23 (3.87)	75.66 (2.42)	75.17 (0.51)
	1 m	0.33 (0.001)	0.32 (0.05)	0.31 (0.05) ^e^*	0.32 (0.002)	0.33 (0.001) ^c^*
LF/HF ratio	2 m	0.33 (0.01)	0.33 (0.01)	0.32 (0.07)	0.33 (0.02)	0.33 (0.02)
	3 m	0.33 (0.01)	0.32 (0.05)	0.31 (0.06)	0.32 (0.04)	0.33 (0.01)

**Table 3:** Survey result for each sub-area in questionnaire items—mean (SD); superscripts indicate significant differences across columns; * *p* < 0.05; ** *p* < 0.01; a—baseline; b—MA (pre-MR); c—MR; d—MA (pre-slide); e—Slide.

	Baseline	MA (Pre-MR)	MR	MA (Pre-Slide)	Slide
	a	b	c	d	e
T-A score	7.57 (2.05) ^d^*	9.5 (3.22) ^c^**	5.9 (1.35) ^b^**	9.6 (3.27) ^a^*^e^**	6.23 (2.16) ^a^*^d^**
D score	7.23 (2.7)	8.0 (2.49) ^c^**	6.2 (0.55) ^b^**	7.93 (2.56) ^e^**	6.37 (0.72) ^d^**
A-H score	6.73 (1.93)	6.77 (1.28) ^c^*	6.1 (0.3) ^b^*	7.13 (1.57) ^e^**	6.17 (0.53) ^d^**
V score	12.07 (3.88)	11.1 (3.36) ^c^*	13.5 (4.65) ^b^*^e^**	10.8 (2.92)	11.27 (3.9) ^c^**
F score	7.23 (3.0) ^c^**	6.97 (2.55) ^c^*	5.2 (1.51) ^a^**^b^*	7.7 (2.55) ^e^**	5.9 (2.41) ^d^**
C score	3.0 (1.41)	3.57 (1.52) ^c^**	2.33 (0.66) ^b^**	3.47 (1.36) ^e^**	2.47 (0.78) ^d^**
TMD score	19.7 (9.98) ^c^**	23.7 (8.4) ^c^**	12.23 (5.99) ^a^**^b^**	25.03 (8.34) ^e^**	15.87 (6.85) ^d^**
Stress level	1.93 (0.83)	2.4 (0.62) ^c^**	1.43 (0.57) ^b^**	2.33 (0.6) ^e^**	1.67 (0.61) ^d^**
